# The role of the neuro-immune-bone axis in osteoporosis: from bone remodeling imbalance to multi-system interactions

**DOI:** 10.3389/fimmu.2026.1911655

**Published:** 2026-08-11

**Authors:** Yupeng Zhang, Junchen Lu, Guojun Deng, Bohan Hu, Zijian Yang, Zhiqiang Li, Xiaofeng Li

**Affiliations:** Department of Osteology, The First Affiliated Hospital of Jiangxi Medical College, Nanchang University, Nanchang, Jiangxi, China

**Keywords:** bone remodeling process, neuro-immune-bone axis, osteoimmunology, osteoporosis, sympathetic nerve, Treg/Th17

## Abstract

Osteoporosis is a systemic metabolic bone disease characterized by decreased bone mass, destruction of bone microstructure, and increased risk of fractures. Traditional views mainly attribute it to an imbalance between osteoblast-mediated bone formation and osteoclast-mediated bone resorption, but increasing evidence suggests that the pathogenesis and progression of osteoporosis are also regulated by complex interactions among the nervous system, immune system, and skeletal system. The neuro-immune-bone axis provides an important framework for integrating the bidirectional regulation between innervation, immune microenvironment, and bone remodeling process. This article systematically reviews the basis of interactions among sensory nerves, sympathetic nerves, immune cells, and bone cells in bone homeostasis, with a focus on the roles of sympathetic nerve activation, sensory neuropeptide imbalance, and immune inflammatory remodeling in the progression of osteoporosis. Furthermore, this article compares the differential imbalance patterns of this axis in postmenopausal osteoporosis, age-related osteoporosis, and secondary osteoporosis, and accordingly discusses its potential significance for disease classification and risk identification. At the therapeutic level, this article further distinguishes between clinically well-established management strategies and mechanistic interventions still in the exploratory stage. The former includes guideline-recommended anti-osteoporosis drugs, exercise, nutritional support, fall prevention, and control of primary diseases; the latter includes strategies such as sympathetic nerve regulation, CGRP-related interventions, Treg/Th17 balance regulation, and macrophage polarization, which are currently mainly based on animal experiments, mechanistic studies, observational evidence, or early translational research and cannot yet be considered routine clinical treatment. Therefore, the neuro-immune-bone axis is currently more suitable as a theoretical framework to explain the heterogeneity of osteoporosis and guide mechanistic research, and its practical value in precise classification, treatment selection, and clinical decision-making still requires further validation through prospective cohort and intervention trials. By integrating existing evidence, this article aims to offer a theoretical foundation for understanding the multi-system interaction mechanisms of osteoporosis, establishing an evidence stratification framework, and exploring future individualized interventions.

## 1 Introduction

Osteoporosis is a systemic skeletal disease characterized by reduced bone mass, deterioration of bone microarchitecture, and increased fracture risk, and is a major cause of disability and decreased quality of life in the elderly population worldwide (1, 2). With the acceleration of population aging, the medical and social burden associated with osteoporotic fragility fractures continues to increase (3). Among these, hip and vertebral fractures are closely related to elevated mortality risk, long-term disability, and consumption of medical resources (4). Traditional views primarily attribute osteoporosis to an imbalance in bone remodeling, where osteoclast-mediated bone resorption exceeds osteoblast-mediated bone formation, leading to decreased bone mass and degradation of bone microarchitecture (5). This “osteoblast-osteoclast imbalance” model has laid the foundation for the diagnosis and treatment of osteoporosis, but it is insufficient to fully explain the significant heterogeneity in bone loss mechanisms and treatment responses across different etiologies, ages, and clinical contexts.

In recent years, an increasing number of studies have suggested that bone homeostasis is not maintained solely by bone cells but is co-regulated by the nervous system, immune system, endocrine system, and the local bone microenvironment (6, 7). Diverse etiological factors, such as menopause, aging, diabetes mellitus, glucocorticoid exposure, chronic inflammatory diseases, and nerve injury, can affect bone metabolism through specific yet intertwined pathways (8). The associated bone loss not only manifests as osteoclast activation or decreased osteoblast function but is also often accompanied by changes in sympathetic nerve activity, imbalance of sensory neuropeptides, remodeling of immune cell function, and sustained release of inflammatory factors (9, 10). These findings suggest that changes in osteoblasts and osteoclasts are more likely the terminal manifestations of the convergence of multiple systemic abnormalities, and focusing solely on bone formation and bone resorption is insufficient to fully reveal the upstream regulatory network of osteoporosis.

On this basis, recent systematic research on the neuro-immune-bone axis has further clarified the multi-level interactions between sensory nerves, sympathetic nerves, neuropeptides, and skeletal stem/progenitor cells, providing important evidence for constructing a theoretical framework of the neuro-immune-bone axis in osteoporosis (11). This axis emphasizes that the nervous system can regulate the bone marrow immune microenvironment and bone cell function through sympathetic nerves, sensory neuropeptides, and central neuroendocrine signals (12); the immune system can influence osteoclastogenesis, osteoblast function, and the bone remodeling process through mechanisms such as inflammatory factors, the receptor activator of nuclear factor-κB ligand/downregulate osteoprotegerin (RANKL/OPG) system, Treg/Th17 balance, and macrophage polarization (13, 14). Reciprocally, osteoblasts, osteoclasts, osteocytes, and bone marrow stromal cells can also influence the local immune status and neural microenvironment through cytokines, chemokines, and metabolic signals (15). Therefore, the neuro-immune-bone axis is not an independent or unidirectional signaling pathway but an integrated summary of neural regulation, osteoimmunology, the bone marrow cell niche, and the bone remodeling network. Based on this framework, osteoporosis can be viewed as a multisystem disease where abnormal neural signals, immune microenvironment imbalance, and bone remodeling disorders mutually amplify each other.

This article will review the role of the neuro-immune-bone axis in osteoporosis. First, it will elaborate on the basis of interactions among innervation, the immune microenvironment, and bone remodeling in bone homeostasis; second, it will analyze key mechanisms such as sympathetic overactivation, imbalance of sensory nerves and neuropeptides, release of inflammatory factors, Treg/Th17 imbalance, and abnormal macrophage polarization; subsequently, it will compare the differential manifestations of this axis in postmenopausal osteoporosis, senile osteoporosis, and secondary osteoporosis; finally, it will summarize potential therapeutic strategies targeting the neuro-immune-bone axis, levels of evidence, and future translational challenges. By integrating basic research, clinical observations, and early translational evidence, this article aims to elucidate the multisystem interaction mechanisms in different osteoporosis phenotypes and provide a theoretical basis for risk stratification, mechanistic subtyping, and individualized intervention.

## 2 Fundamentals of neuro-immune-bone interactions in bone homeostasis

### 2.1 Innervation characteristics of bone tissue

Bone tissue is not a passive mechanical support structure but an active organ co-regulated by nerves, blood vessels, immune cells, and bone cells (16). Different types of nerve fibers are distributed in the periosteum, bone marrow cavity, trabecular bone surface, and around bone blood vessels, among which sensory nerves and sympathetic nerves are the main neural components involved in bone homeostasis regulation. These nerve fibers not only participate in pain perception and vasomotor regulation but also directly or indirectly modulate the activity of osteoblasts, osteoclasts, bone cells, bone marrow stromal cells, and immune cells by releasing neurotransmitters and neuropeptides, thereby modulating the bone remodeling process (17, 18).

Sensory nerves primarily regulate the local bone microenvironment by releasing neuropeptides such as calcitonin gene-related peptide (CGRP) and substance P (SP). CGRP exerts a bone-protective effect, promoting osteoblast proliferation, differentiation, and mineralization, while partially inhibiting osteoclastogenesis and bone resorption (19, 20). Additionally, sensory nerves constitute a critical component of the skeletal stem cell (SSC) niche, and their functional integrity is essential for maintaining SSC self-renewal and osteogenic differentiation potential (21). Recently, Li et al. systematically summarized the research progress on the nerve-bone axis, pointing out that the role of bone innervation is not limited to pain perception and vascular regulation but also involves the synergistic regulation of skeletal stem/progenitor cell fate, neuro-vascular coupling, osteogenesis and osteoclast activity, and bone regeneration processes by sensory nerves, sympathetic nerves, and their neurotransmitters and neuropeptides (22). This understanding further indicates that nerve fibers and their related signals are important functional components of the skeletal stem/progenitor cell niche and the bone remodeling microenvironment, rather than merely serving sensory afferent or vasomotor regulatory functions. In contrast, the role of SP is more context-dependent, as it can participate in neurogenic inflammation and pain responses while also affecting bone remodeling by regulating local immune cells and vascular responses (23, 24). Therefore, sensory nerves are not simply sensory afferent structures but important regulatory hubs connecting mechanical stimulation, neuropeptide release, immune regulation, and bone formation.

Sympathetic nerve fibers primarily participate in bone metabolism regulation by releasing signaling molecules such as norepinephrine (NE) and neuropeptide Y (NPY). NE can act on β2-adrenergic receptors (β2-AR) on the surface of osteoblasts, osteoclasts, and some immune cells, affecting osteogenic differentiation, osteoclastogenesis, and local inflammatory responses (25, 26). However, the effect of sympathetic nerves on bone metabolism is complex and non-linear, and its impact may be influenced by age, hormonal status, local inflammation, mechanical load, and underlying pathological conditions. Most studies suggest that excessive sympathetic activation tends to promote bone resorption and inhibit bone formation, thereby driving bone remodeling toward a negative balance (27). Nevertheless, the influence of sympathetic nerves on bone metabolism is not entirely linear, and its effects may be modulated by age, hormonal status, local inflammation, mechanical load, and disease background. Therefore, sensory nerves and sympathetic nerves do not independently regulate bone metabolism; instead, they cooperatively maintain bone homeostasis through neuropeptides, neurotransmitters, blood vessels, and the bone marrow cell niche, and their relative activity changes may determine the dynamic balance between local bone formation and bone resorption.

Bone innervation exhibits dynamic plasticity and can change with aging, hormonal fluctuations, inflammatory states, nerve injury, and metabolic abnormalities (28). Sensory nerve degeneration, decreased CGRP expression, increased sympathetic nerve activity, or abnormal nerve fiber distribution may alter the local bone microenvironment and affect the balance between bone formation and bone resorption (29). Therefore, the dynamic changes in bone innervation are an important basis for understanding the imbalance of the neuro-immune-bone axis.

### 2.2 Bone immune microenvironment and bone remodeling

The bone immune microenvironment, composed of various immune cells, cytokines, chemokines, and bone marrow stromal components, serves as a critical foundation for maintaining bone remodeling balance. Immune cells such as macrophages, T cells, B cells, and dendritic cells can regulate osteoclast differentiation, osteoblast function, and bone cell activity through direct cell contact or secretion of soluble factors (30, 31). Under physiological conditions, bidirectional communication between immune cells and bone cells maintains the dynamic balance between bone resorption and bone formation; however, in the context of inflammation, aging, or metabolic disorders, dysregulation of the immune microenvironment may promote increased bone resorption and inhibited bone formation (32, 33).

Macrophages are important regulatory cells in the bone immune microenvironment, and their polarization state significantly impacts bone remodeling. Classically activated M1-type macrophages secrete pro-inflammatory factors such as TNF-α, IL-1β, and IL-6, promoting local inflammation and osteoclastogenic responses; alternatively activated M2-type macrophages secrete more anti-inflammatory or repair-related factors such as IL-10 and TGF-β, contributing to inflammation resolution and tissue repair (34, 35). Additionally, bone macrophages (OsteoMacs), as tissue-resident macrophages on the bone surface or in the bone marrow microenvironment, participate in bone formation and repair by regulating osteoblast activity and local immune status (36, 37). Therefore, macrophages are not merely inflammatory effector cells but important regulatory nodes connecting immune responses, bone formation, and bone resorption.

T cell subsets are also important components of bone immune regulation. Among them, the balance between T helper 17 cells (Th17) and regulatory T cells (Treg) is significant for bone remodeling. Th17 cells promote RANKL expression and osteoclast differentiation by secreting IL-17, thereby enhancing bone resorption; Treg cells inhibit osteoclast formation and maintain immune homeostasis by secreting immunosuppressive factors such as IL-10 and TGF-β (38, 39). In an inflammatory environment, a shift in the Treg/Th17 balance toward a pro-inflammatory direction can drive the bone microenvironment from homeostasis toward enhanced bone resorption (40). B cells and T cells can also express RANKL, directly participating in the osteoclastogenesis process (41).

The RANKL/OPG system is one of the important molecular interfaces between the immune system and bone remodeling. RANKL binding to RANK on the surface of osteoclast precursors drives osteoclast differentiation and activation; osteoprotegerin (OPG), as a decoy receptor for RANKL, can block RANKL-RANK binding and inhibit osteoclastogenesis (42). Under inflammatory conditions, factors such as TNF-α, IL-1β, IL-6, and IL-17 can upregulate RANKL expression or alter the RANKL/OPG ratio, thereby promoting osteoclast-mediated bone resorption (43). Therefore, the RANKL/OPG system is not only a core pathway for osteoclast differentiation but also a key molecular node where neural signals and immune inflammation converge to influence the bone remodeling process.

### 2.3 Neuro-immune-bone homeostasis regulatory mechanisms

The core of the neuro-immune-bone axis lies in the bidirectional or even multi-directional regulatory relationships among the nervous system, immune system, and bone tissue. First, the nervous system can influence bone remodeling by regulating the immune microenvironment. NE released by sympathetic nerves can act on β2-AR on immune cells and bone cells, altering pro-inflammatory factor release, RANKL/OPG expression, and osteoclastogenesis (44). Under pathological sympathetic excitation, this pathway may amplify local inflammatory responses and bone resorption processes. Conversely, CGRP released by sensory nerves can, to some extent, inhibit pro-inflammatory responses, regulate macrophage polarization, and support osteoblast-related cell functions (45). Therefore, sympathetic and sensory nerves often exhibit a reciprocal regulatory balance in the bone microenvironment.

Second, the immune system can also modulate neural function and the local neural microenvironment of bone. Pro-inflammatory factors such as TNF-α, IL-1β, and IL-6 can not only directly promote osteoclastogenesis but also influence sympathetic nerve tone, sensory nerve sensitivity, and neuropeptide release status (46, 47). Chronic inflammatory environments can enhance neurogenic inflammatory responses, alter bone tissue pain sensitivity and local vascular responses, and further affect bone remodeling. Thus, a positive feedback loop can form between neural abnormalities and immune inflammation, keeping the local bone microenvironment in a prolonged state of pro-osteoclastogenic activity and impaired repair (48).

**Figure 1:**
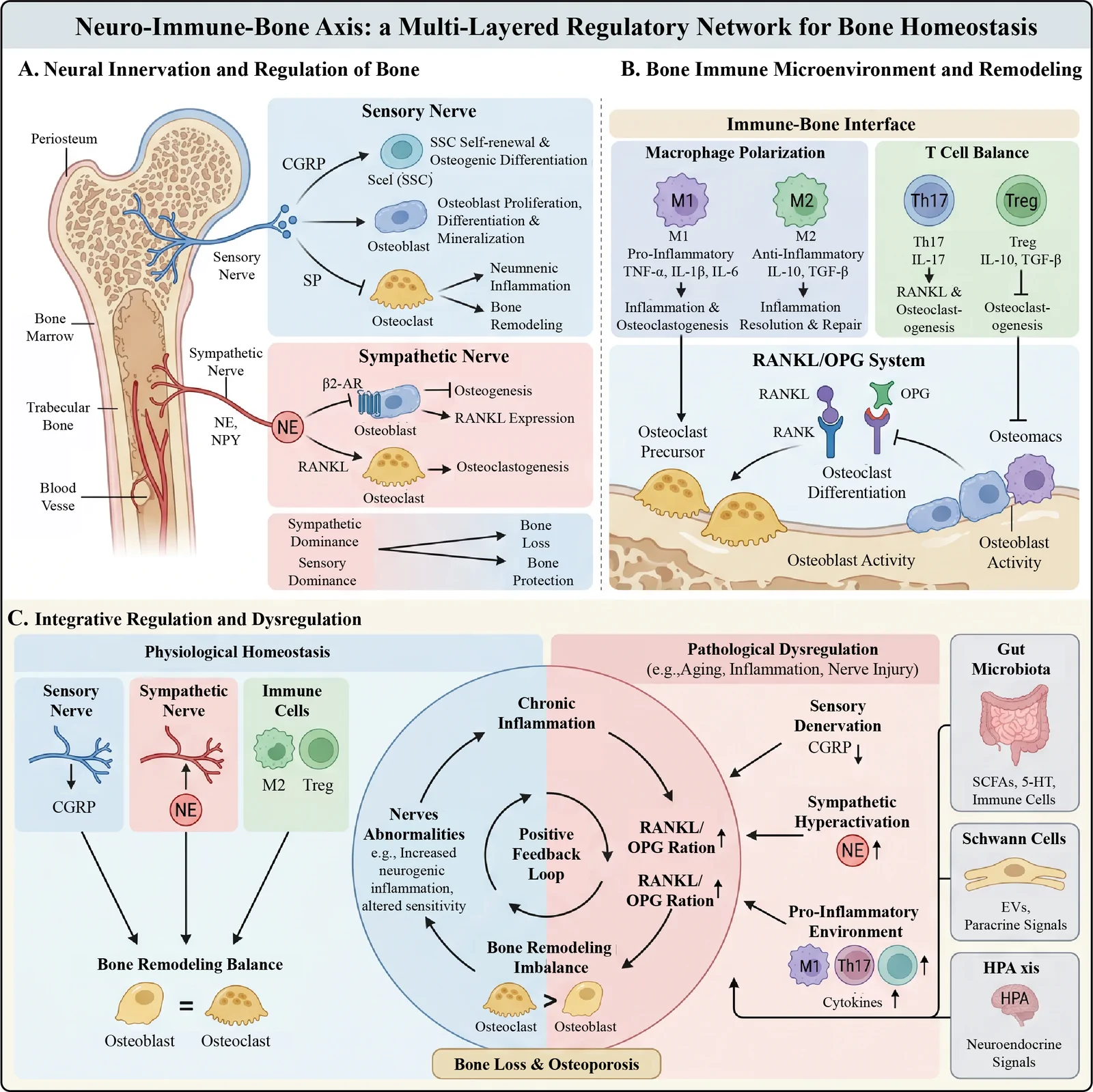
Neuro-immune-bone axis maintains bone homeostasis and its pathological imbalance. **(A)** Sensory nerves release CGRP and SP, while sympathetic nerves release NE and NPY, jointly regulating skeletal stem/progenitor cells, osteoblasts, osteoclasts, and the bone marrow microenvironment. **(B)** Macrophage M1/M2 polarization, Treg/Th17 balance, and the RANKL/OPG system collectively maintain the balance between bone formation and bone resorption. **(C)** Aging, chronic inflammation, or nerve injury can lead to sensory nerve degeneration, sympathetic nerve activation, and immune inflammation amplification, ultimately resulting in bone remodeling imbalance and osteoporosis. β2-AR, β2-adrenergic receptor; CGRP, calcitonin gene-related peptide; NE, norepinephrine; NPY, neuropeptide Y; OPG, osteoprotegerin; RANKL, receptor activator of nuclear factor-κB ligand; SP, substance P; SSC, skeletal stem cell.

Additionally, the neuro-immune-bone axis is influenced by various peripheral regulatory modules. Gut microbiota can affect bone metabolism through short-chain fatty acids, serotonin metabolism, intestinal barrier function, and immune cell differentiation, and participate in central nervous and peripheral immune regulation via the gut-brain signal (49). Peripheral nerve support cells such as Schwann cells can also influence osteoclastogenesis and bone repair-related pathways through extracellular vesicles and paracrine signals (50). Meanwhile, neuroendocrine systems such as the hypothalamic-pituitary-target gland axis can integrate peripheral metabolism, inflammation, and stress signals, exerting systemic effects on bone metabolism (51). Therefore, the neuro-immune-bone axis is not a single linear pathway but a multi-level regulatory network involving neurotransmitters, immune cells, bone cells, gut microbiota, and neuroendocrine signals. The maintenance of homeostasis depends on the dynamic balance among neural signals, immune responses, and bone remodeling; once this balance is disrupted by aging, hormonal deficiency, chronic inflammation, metabolic abnormalities, or nerve injury, it may contribute to the onset and progression of osteoporosis (**Figure 1**).

## 3 Neurological abnormalities and osteoporosis

### 3.1 Sympathetic overactivation and bone loss

Abnormal sympathetic nerve activity, particularly persistent overactivation, may be a significant upstream regulatory factor in the development and progression of osteoporosis. Bone tissue is richly innervated by sympathetic nerves, and the NE released can act on β2-AR on the surface of osteoblasts, osteoclasts, and some immune cells, thereby participating in the regulation of bone remodeling (52). Under conditions such as chronic stress, nerve injury, inflammation, or hormonal deficiency, increased sympathetic tone can activate the β2-AR–cAMP–PKA signaling pathway, inhibiting osteoblast proliferation, differentiation, and bone formation, while promoting osteoclastogenesis and bone resorption, ultimately tipping the balance of bone remodeling toward net loss (53). A recent comprehensive review on the neuro-osseous axis further indicates that sympathetic nerve signals not only directly act on bone cells but may also connect central nervous system regulation, changes in the bone marrow microenvironment, and bone remodeling imbalance; their specific effects are influenced by age, hormonal status, mechanical load, local inflammation, and disease context (22). Therefore, the regulation of bone metabolism by the sympathetic nervous system should be understood as a context-dependent network effect rather than a single, linear bone-resorptive process.

Animal studies and some clinical observations suggest that increased sympathetic nerve activity is associated with decreased bone mass and increased fracture risk. For example, long-term psychological stress, depressive states, or the use of β2-AR agonists may be linked to lower bone mineral density and higher fracture risk (54, 55). Conversely, beta-blockers, particularly some non-selective beta-blockers, have shown a trend towards improved bone density or reduced fracture risk in animal models and observational studies (56). However, these results are mainly hypothesis-generating and may be confounded by underlying cardiovascular diseases, indication bias, concomitant medications, and lifestyle factors; thus, they are insufficient to prove that beta-blockers have a direct anti-osteoporotic effect.

At the molecular level, sympathetic nerve signals can upregulate the expression of RANKL, OPG expression, and increase the RANKL/OPG ratio, thereby enhancing osteoclast-mediated bone resorption (57). Simultaneously, sympathetic activation may inhibit pro-osteogenic signals such as Wnt/β-catenin, further weakening bone formation capacity. These mechanisms together constitute a dual effect of “enhanced bone resorption—suppressed bone formation,” suggesting that the sympathetic nervous system may amplify bone remodeling imbalance by simultaneously regulating bone cells and the bone marrow microenvironment. Although these mechanisms provide a theoretical basis for targeting sympathetic nerve signals, current clinical evidence is insufficient to support beta-blockers as a routine treatment for osteoporosis, nor can they replace guideline-recommended therapies such as bisphosphonates, denosumab, or bone-forming agents (58). Therefore, beta-blockers should currently be considered a potential candidate for drug repurposing within the sympathetic nervous system-bone axis, rather than an established anti-osteoporotic treatment strategy; their true skeletal benefits, applicable populations, and safety require further validation through prospective clinical trials.

### 3.2 Sensory nerve and neuropeptide imbalance

Sensory nerves and the neuropeptides they release are not only involved in pain transmission but also contribute to maintaining homeostasis of the local bone microenvironment by regulating bone cells, immune cells, blood vessels, and skeletal stem/progenitor cells. CGRP and SP are representative neuropeptides released from sensory nerve endings, with distinct roles in bone metabolism (59). In most animal and *in vitro* studies, CGRP exhibits a bone-protective tendency, promoting osteoblast proliferation, differentiation, and mineralization, while inhibiting osteoclast differentiation and bone resorption to some extent (60). In ovariectomy (OVX)-induced osteoporosis animal models, the density of CGRP-positive nerve fibers and CGRP expression levels in bone tissue decrease, often accompanied by reduced bone mineral density and deterioration of bone microarchitecture, suggesting that sensory nerve dysfunction may be involved in bone loss. However, current evidence for CGRP primarily comes from animal models and local bone repair studies, with limited direct clinical evidence from osteoporosis patients (61, 62). Therefore, decreased CGRP is more suitable as a candidate mechanism linking sensory nerve degeneration and bone loss, rather than a fully validated clinical causal pathway. A recent comprehensive review on the neuro-osseous axis further highlights that sensory nerve regulation of the skeleton is not limited to the isolated effects of CGRP or SP, but also involves coordinated regulation of neuropeptide release, angiogenesis, skeletal stem/progenitor cell recruitment and differentiation, and the bone regeneration microenvironment (63). Thus, sensory nerve dysfunction may simultaneously affect bone formation, bone repair, and neuro-vascular coupling, with outcomes depending on the type of neuropeptide, disease state, and tissue repair stage.

Compared to CGRP, the role of SP in bone metabolism is more context-dependent. SP can mediate neurogenic inflammation, immune cell recruitment, and local inflammatory responses through the neurokinin-1 receptor (NK-1R) (23). In some inflammatory or bone loss models, SP may promote the release of pro-inflammatory factors, enhance osteoclastogenesis, and exacerbate bone resorption (64). However, SP does not always act as a pro-resorptive signal; in specific injury stages or repair microenvironments, it may also produce different effects by regulating local blood flow, pain perception, vascular responses, and tissue repair (65). Therefore, the role of SP in bone metabolism should be interpreted in conjunction with dosage, timing, and the local microenvironment, rather than simply being categorized as a unidirectional pro-inflammatory or pro-osteoclastic factor. Sensory nerve dysfunction can occur in various pathological contexts such as diabetic neuropathy, spinal cord injury, and aging, and may aggravate bone microenvironment disturbances through decreased CGRP, abnormal SP signaling, and local neuro-immune response imbalance (66–68). These changes suggest that sensory nerve degeneration in different disease contexts may affect bone remodeling through a composite mechanism of “reduced bone-protective neuropeptides—neurogenic inflammation imbalance—impaired neurovascular support.”

In recent years, intervention strategies based on CGRP or sensory nerve regeneration have shown potential in osteoporotic fracture repair and local bone regeneration models. For example, a composite hydrogel capable of sequentially releasing stromal cell-derived factor-1 (SDF-1) and CGRP can promote callus mineralization, remodeling, and neurovascular network regeneration in osteoporotic fracture models (69). These results suggest that locally restoring sensory nerve signals or rebuilding neuro-vascular-bone coupling may help improve the fracture repair microenvironment, but they cannot yet be extrapolated to infer systemic anti-osteoporotic efficacy. Current research is mainly limited to animal models, biomaterials, and local delivery systems, lacking evidence on human efficacy, long-term safety, and anti-fracture outcomes. Therefore, CGRP delivery, sensory nerve regeneration, and neuro-vascular coupling reconstruction should currently be positioned as preclinical research strategies in the field of osteoporotic fracture repair or local bone regeneration, rather than established systemic anti-osteoporotic treatments. Their effective doses, delivery methods, therapeutic windows, off-target effects, systemic safety, and long-term anti-fracture benefits require further validation.

### 3.3 Neurotransmitters and bone metabolism

In addition to NE, CGRP, and SP, various neurotransmitters and neuropeptides are involved in the regulation of bone metabolism, with serotonin (5-HT) being the most prominent example. The effect of 5-HT on bone metabolism is distinctly source-dependent. Central 5-HT, primarily synthesized by tryptophan hydroxylase 2, can indirectly promote bone formation by regulating sympathetic output through the hypothalamus (70, 71). In contrast, peripheral 5-HT, mainly derived from intestinal enterochromaffin cells and synthesized by Tph1, acts as a hormone-like signal on the 5-HT1B receptor on the surface of osteoblasts, inhibiting osteoblast proliferation and bone formation via PKA-cAMP-CREB-related pathways (72). Therefore, central 5-HT and peripheral 5-HT may have opposing effects on bone metabolism, and this dual effect is a key entry point for understanding the regulation of bone homeostasis by neurotransmitters.

Based on the mechanism by which peripheral 5-HT inhibits bone formation, Tph1 inhibitors such as LP533401 have been explored in experimental studies as candidate strategies to enhance bone formation. Notably, such strategies remain primarily in the experimental stage, and their long-term safety, target populations, and true anti-fracture benefits have not been fully validated clinically (73). Therefore, modulating 5-HT signaling is more suitable as a mechanistic target in neuro-osseous axis research rather than a mature anti-osteoporotic treatment regimen at this stage.

**Figure 2:**
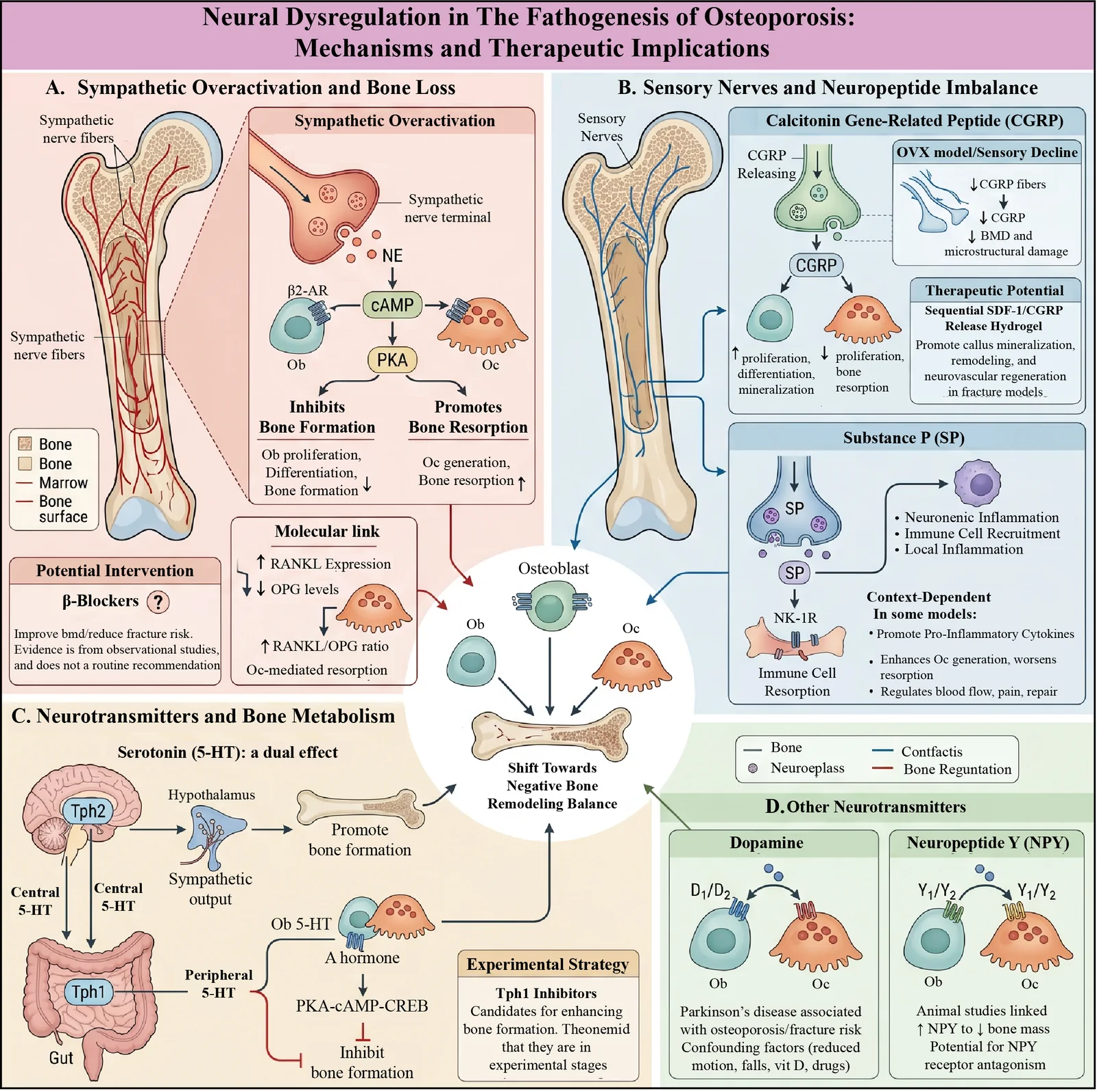
Major mechanisms of neural regulation abnormalities involved in osteoporosis. **(A)** Sympathetic activation can increase the RANKL/OPG ratio via NE-β2-AR-cAMP-PKA signaling and inhibit pro-osteogenic signals, leading to enhanced bone resorption and suppressed bone formation. **(B)** Sensory nerve dysfunction and decreased CGRP can impair osteogenesis, neuro-vascular coupling, and bone repair; SP participates in neurogenic inflammation through NK-1R, with context-dependent effects. **(C)** Neurotransmitters such as 5-HT, dopamine, and NPY can influence bone remodeling through central or peripheral pathways. Beta-blockers, CGRP delivery, and neurotransmitter modulation are currently still exploratory or preclinical research directions. Abbreviations: β2-AR, β2-adrenergic receptor; CGRP, calcitonin gene-related peptide; 5-HT, serotonin; NE, norepinephrine; NK-1R, neurokinin-1 receptor; NPY, neuropeptide Y; OPG, osteoprotegerin; PKA, protein kinase A; RANKL, receptor activator of nuclear factor-κB ligand; SP, substance P.

Dopamine and NPY may also be involved in the regulation of bone metabolism, but the relevant evidence is relatively limited. Dopamine can affect osteoblast and osteoclast function through D1/D2 receptors; the increased risk of osteoporosis and fractures in Parkinson’s disease patients also suggests an association between degeneration of the dopaminergic system and abnormal bone metabolism (74, 75). However, these clinical associations may be confounded by factors such as reduced physical activity, increased fall risk, vitamin D deficiency, and medication use, and thus cannot be solely attributed to dopamine deficiency. NPY, primarily derived from sympathetic nerves and central neurons, can affect osteoblast activity and bone resorption through Y1/Y2 receptors (76). Some animal studies suggest that enhanced NPY signaling is associated with decreased bone mass, while NPY receptor antagonism may have bone-protective effects, but its clinical translation is still in the early stages (77). Overall, neurotransmitters such as 5-HT, dopamine, and NPY may collectively contribute to the regulation of bone homeostasis, but their clinical utility in osteoporosis classification and treatment warrants further validation (**Figure 2**).

## 4 Immune imbalance mediates the progression of osteoporosis

### 4.1 Inflammatory bone loss

Inflammatory bone loss is an important pathological process in osteoporosis and various bone metabolic diseases, characterized by persistently elevated pro-inflammatory cytokines, enhanced osteoclastogenesis, and suppressed bone formation (78). Among numerous inflammatory factors, TNF-α, IL-1β, and IL-6 are key mediators linking immune activation to increased bone resorption. TNF-α promotes osteoclast precursor differentiation by activating nuclear factor κB (NF-κB) and mitogen-activated protein kinase (MAPK) signaling pathways, and synergistically amplifies osteoclast effects with RANKL (79, 80). IL-1β upregulates RANKL expression in osteoblasts and immune cells while inhibiting OPG production, thereby increasing the RANKL/OPG ratio and promoting osteoclast formation and activation (81). IL-6 contributes to osteoclastogenesis and exacerbated bone resorption under chronic inflammatory conditions via mechanisms involving the JAK/STAT3 pathway (82). Therefore, the sustained release of pro-inflammatory factors shifts the bone microenvironment from homeostasis to bone resorption dominance, forming the molecular basis of immune-mediated bone loss.

Chronic inflammatory diseases provide important references for understanding inflammatory bone loss. In rheumatoid arthritis (RA), signals such as TNF-α, IL-1β, IL-6, and RANKL not only contribute to local bone erosion but also affect systemic bone mineral density (83). Alveolar bone destruction in periodontitis is similarly closely related to local inflammatory factor release, osteoclast activation, and RANKL/OPG imbalance (84). Additionally, lipopolysaccharide (LPS)-induced inflammatory models are commonly used to simulate inflammatory bone loss, with related studies showing that they promote systemic inflammation and enhanced bone resorption, leading to impaired trabecular bone structure (85). It should be noted that RA, periodontitis, and LPS models provide important mechanistic insights into inflammatory bone loss, but their underlying pathologies are not entirely equivalent to primary osteoporosis; therefore, relevant conclusions should be generalized with caution.

Clinically, elevated systemic inflammation is associated with decreased bone mineral density and increased fracture risk. For example, elevated C-reactive protein is often regarded as an indirect indicator of chronic low-grade inflammatory burden (86). Anti-inflammatory therapy in inflammatory diseases such as RA can exert a protective effect on bone mineral density by controlling disease activity, reducing inflammatory factor release, and decreasing RANKL-driven osteoclast effects (87). However, this benefit more reflects “indirect bone protection after controlling inflammatory disease” and does not imply that anti-inflammatory drugs can be directly used as routine treatment for general osteoporosis patients. Therefore, inflammatory bone loss is more suitable as an important mechanistic framework for identifying inflammation-dominant osteoporosis or secondary bone loss, and its clinical application still requires assessment based on the type of primary disease, inflammatory burden, and bone turnover status.

### 4.2 Treg/Th17 imbalance

Regulatory T cells (Treg) and T helper 17 cells (Th17) are a pair of key immune subsets in bone immunoregulation, and their balance significantly impacts bone homeostasis. Treg cells primarily inhibit osteoclast precursor differentiation and maintain immune tolerance by secreting anti-inflammatory factors such as IL-10 and TGF-β (88, 89). Conversely, Th17 cells secrete pro-inflammatory factors such as IL-17A, IL-17F, and IL-22, inducing RANKL expression in osteoblasts, stromal cells, or immune cells, thereby promoting osteoclastogenesis and bone resorption (90). IL-17A also promotes the release of pro-inflammatory factors such as TNF-α and IL-1β from macrophages and fibroblasts, further amplifying local inflammatory responses and osteoclast activity (91). Therefore, the Treg/Th17 balance can be regarded as a critical node in the immune system’s regulation of the switch between bone resorption and bone protection.

Multiple studies suggest that in osteoporosis patients or related animal models, the Treg/Th17 balance may shift toward Th17 dominance, manifested as increased Th17 cells, decreased Treg cells, or reduced Treg function (92). This immune shift can reduce immune tolerance, enhance pro-inflammatory factor release, and promote osteoclast activation through RANKL-related pathways, thereby contributing to bone loss. Studies in senescence-accelerated mouse models have shown that bone marrow mesenchymal stem cell-derived exosomes can regulate the Treg/Th17 balance through pathways such as miR-21-5p/SKP2/FoxO1 and improve age-related bone loss (93). Inflammatory bone destructive diseases such as RA also support a close association between Treg/Th17 imbalance and local bone erosion; however, given that their inflammatory milieu differs from that of primary osteoporosis, these findings should be interpreted as extrapolated mechanistic evidence (94). These findings support the biological plausibility of Treg/Th17 imbalance in inflammation-related bone loss, but are insufficient to establish it as a universal therapeutic target for all osteoporosis patients.

From a translational perspective, restoring Treg/Th17 balance is an exploratory immunomodulatory direction with mechanistic rationale, but no mature clinical intervention for osteoporosis has been established. Low-dose IL-2, IL-17 pathway inhibition, mesenchymal stem cell exosomes, and gut microbiota modulation have shown potential for regulating Treg/Th17 balance in some inflammatory diseases or preclinical studies (95). However, the evidence primarily comes from disease-specific populations, animal models, or early translational studies, lacking high-quality clinical trials with endpoints such as fragility fractures, long-term bone mineral density changes, or safety in osteoporosis patients. Additionally, the clinical use of IL-17 pathway inhibitors in certain inflammatory diseases is primarily based on their role in controlling disease activity, and their potential bone-protective effects cannot be directly extrapolated as an independent anti-osteoporosis indication (96).

In the future, peripheral blood Treg/Th17 ratio, IL-17A, IL-10, and RANKL/OPG ratio could serve as exploratory immunophenotyping indicators to identify osteoporosis patients with a putative inflammation-dominant phenotype, providing a basis for subsequent phenotype-enriched intervention studies. Therefore, Treg/Th17 modulation is currently more suitable as a research tool for immunophenotype identification, mechanism validation, and clinical trial population enrichment, rather than a routine osteoporosis treatment option.

### 4.3 Macrophage polarization and bone remodeling

Macrophages are core effector cells in the bone immune microenvironment, exhibiting high functional plasticity. Under different microenvironmental stimuli, macrophages can differentiate into the classically activated M1 phenotype or the alternatively activated M2 phenotype (97). M1-type macrophages are typically induced by pro-inflammatory signals such as LPS, interferon-γ (IFN-γ), and TNF-α, characterized by high expression of inducible nitric oxide synthase (iNOS) and CD86, and secretion of pro-inflammatory factors such as TNF-α, IL-1β, and IL-6 (98, 99). These factors promote osteoclastogenesis, enhance bone resorption, and inhibit osteoblast function. M2-type macrophages are mostly induced by signals such as IL-4 and IL-13, expressing CD206 and arginase 1, and secreting anti-inflammatory and repair-related factors such as IL-10 and TGF-β, contributing to inflammation resolution, tissue repair, and bone formation (100). Therefore, the M1/M2 polarization balance is crucial for maintaining bone immune microenvironment stability and bone remodeling homeostasis.

In bone loss states such as osteoporosis, the bone marrow immune microenvironment often exhibits a pro-inflammatory shift, with an increased M1/M2 ratio or insufficient M2 repair programs. OVX-induced osteoporosis models show an increased proportion of M1-type macrophages in the bone marrow, associated with decreased bone mineral density and deterioration of trabecular bone structure (101). In diabetes-related delayed bone healing models, persistent M1-type macrophages dominance and insufficient M2-type macrophages response are also observed, suggesting that the inflammatory microenvironment not only promotes bone resorption but may also hinder bone repair and regeneration (102). Therefore, abnormal macrophage polarization may be an important immune link connecting chronic inflammation, osteoclast activation, and impaired bone repair. However, polarization states, inducing factors, and skeletal outcomes vary across different models, and these findings still require further validation in human osteoporotic tissues and prospective clinical studies.

Regulating macrophage polarization is an important preclinical direction in current osteoporosis immune microenvironment research, but standardized strategies for routine clinical treatment have not yet been developed. Some preclinical studies have shown that IL-4-loaded scaffolds or hydrogels can induce an M2-like repair phenotype and improve bone repair in bone defect models (103, 104). miRNAs and nanomaterials have also been used to regulate the M1/M2 balance; for example, certain nanoplatforms can improve trabecular bone structure and bone mineral density in OVX models by clearing reactive oxygen species and promoting M1-to-M2 conversion (105, 106). However, such studies primarily evaluate bone defect repair, local inflammation alleviation, or bone microstructure changes in animals, lacking standardized formulations, stable pharmacokinetic parameters, long-term toxicity assessments, and human trials with fragility fractures as endpoints. There is also a lack of unified standards among different materials, delivery routes, and polarization markers, limiting result comparison and clinical translation.

Notably, macrophage polarization interventions also present potential therapeutic paradoxes. For example, PPARγ agonists can promote M2 polarization and have anti-inflammatory effects, but some PPARγ-related drugs may inhibit osteogenic differentiation or increase fracture risk, so “promoting M2 polarization” cannot simply be regarded as an absolutely beneficial bone protection strategy (107). This suggests that when evaluating macrophage-targeting strategies, attention should be paid not only to changes in inflammatory markers but also to osteogenesis, osteoclast activity, bone quality, and long-term safety.

**Figure 3:**
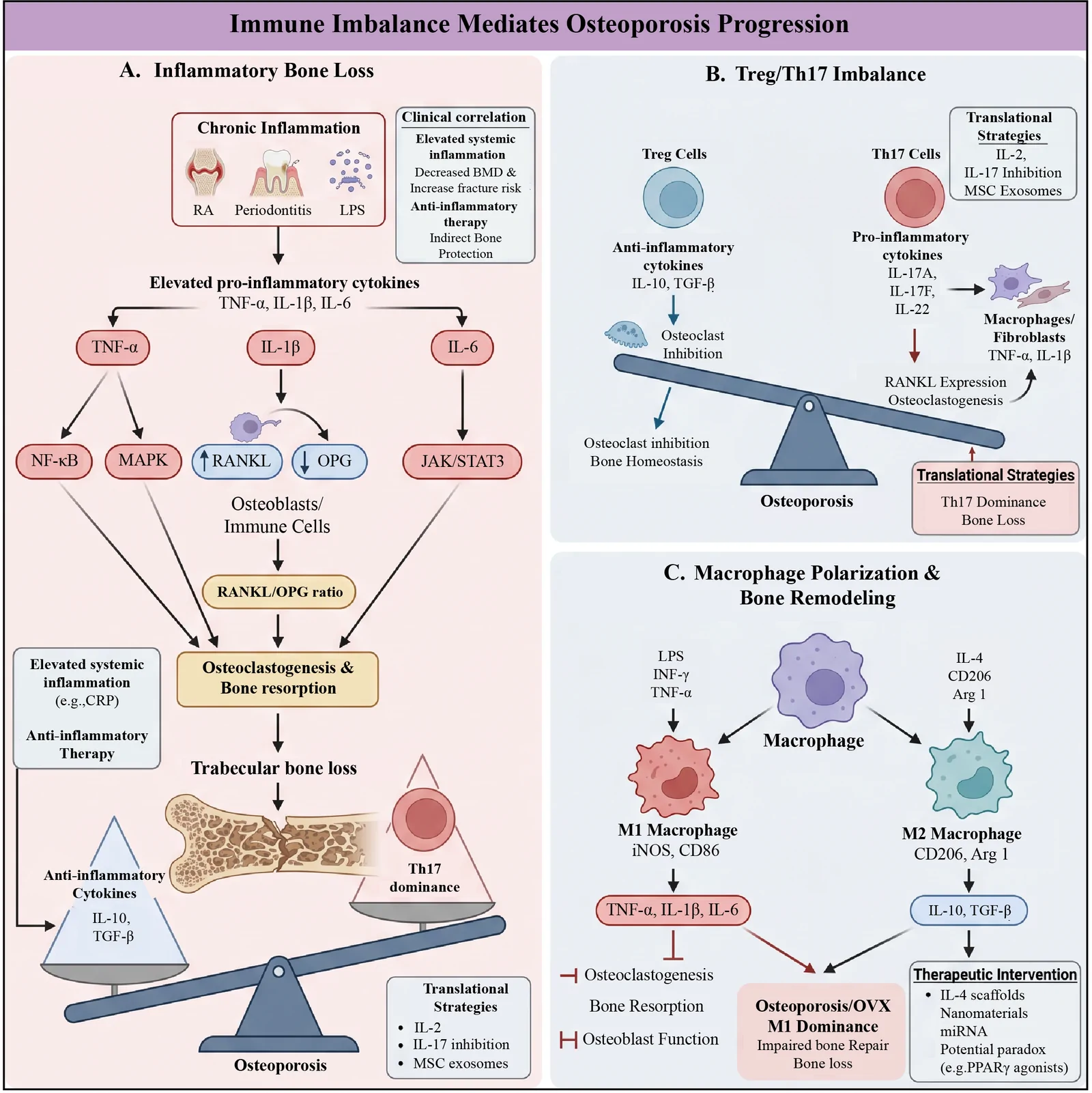
Mechanisms of immune imbalance mediating osteoporosis progression. **(A)** Pro-inflammatory factors such as TNF-α, IL-1β, and IL-6 increase the RANKL/OPG ratio via the NF-κB, MAPK, and JAK/STAT3 pathways, promoting osteoclast formation and bone resorption. **(B)** A decrease in Treg cells and an increase in Th17 cells lead to an imbalance between IL-10, TGF-β, and IL-17A, further amplifying inflammation and osteoclast responses. **(C)** An imbalance in macrophage M1/M2 polarization affects inflammation resolution, bone resorption, and tissue repair. Treg/Th17 modulation and macrophage polarization interventions are currently primarily exploratory or preclinical strategies. Abbreviations: IL, interleukin; JAK, Janus kinase; MAPK, mitogen-activated protein kinase; NF-κB, nuclear factor κB; OPG, osteoprotegerin; RANKL, receptor activator of nuclear factor κB ligand; STAT3, signal transducer and activator of transcription 3; TGF-β, transforming growth factor β; Th17, T helper 17 cells; TNF-α, tumor necrosis factor α; Treg, regulatory T cells.

Overall, M1/M2 polarization regulation should currently be positioned as a direction for mechanism validation, biomaterial development, and local bone repair research, rather than a recommended immunotherapy for osteoporosis. Future research needs to clarify macrophage functional profiles in different osteoporosis subtypes, optimal intervention windows, delivery methods, and off-target risks, and verify their relationships with bone turnover, bone mineral density, and fracture outcomes through standardized human studies (**Figure 3**).

## 5 The role of the neuro-immune-bone axis in different types of osteoporosis

**Figure 4:**
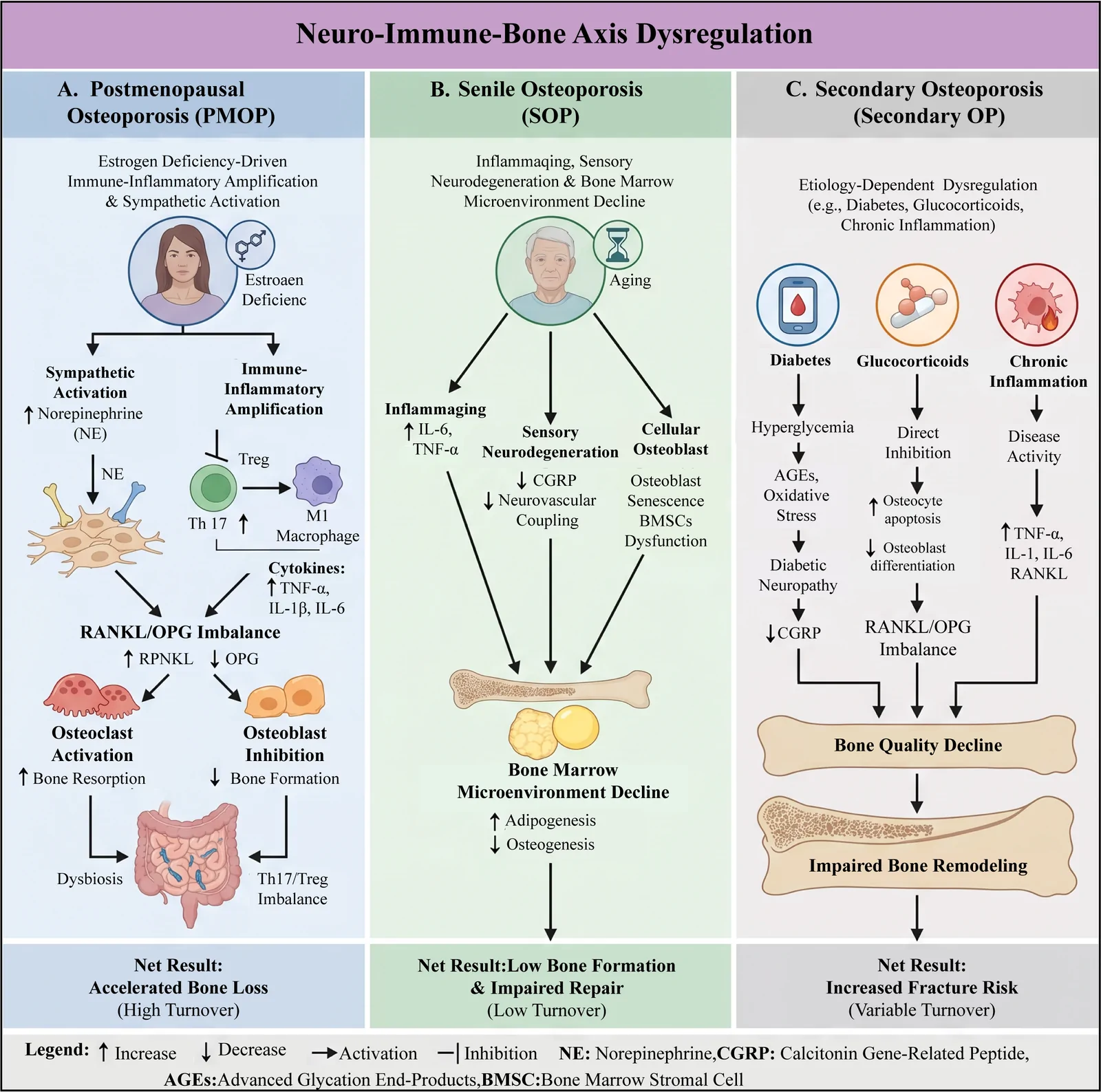
Patterns of neuro-immune-bone axis imbalance in different types of osteoporosis. **(A)** Postmenopausal osteoporosis is mainly characterized by estrogen deficiency, sympathetic nerve activation, Treg/Th17 imbalance, and an elevated RANKL/OPG ratio. **(B)** Senile osteoporosis is primarily characterized by inflammatory aging, sensory nerve degeneration, decreased CGRP, and reduced osteogenesis and repair capacity. **(C)** The mechanism of secondary osteoporosis is etiology-dependent and can be driven by metabolic abnormalities, neuropathy, glucocorticoid-related osteogenesis inhibition, or chronic inflammation. Abbreviations: CGRP, calcitonin gene-related peptide; OPG, osteoprotegerin; RANKL, receptor activator of nuclear factor-κB ligand; Th17, T helper cell 17; Treg, regulatory T cell.

Although different types of osteoporosis ultimately manifest as decreased bone mass, destruction of bone microstructure, and increased fracture risk, their upstream driving factors and patterns of neuro-immune-bone axis imbalance are distinct. Postmenopausal osteoporosis is more characterized by immune inflammatory amplification and sympathetic nerve activation driven by estrogen deficiency (108); senile osteoporosis is characterized predominantly by inflammatory aging, sensory nerve degeneration, osteoblast senescence, and degeneration of the bone marrow microenvironment (109); secondary osteoporosis is often driven by diabetes mellitus, glucocorticoid exposure, chronic inflammatory diseases, or other underlying conditions (110). Therefore, analyzing different types of osteoporosis from the perspective of the neuro-immune-bone axis elucidates their clinical heterogeneity and provides a theoretical basis for stratified therapeutic interventions (**Figure 4**).

### 5.1 Postmenopausal osteoporosis

Postmenopausal osteoporosis (PMOP) is the most common type of primary osteoporosis in women after menopause, with the core trigger being a rapid decline in estrogen levels. Estrogen not only directly acts on osteoblasts, osteoclasts, and osteocytes but also affects bone remodeling by regulating sympathetic nerve activity and the immune microenvironment (111, 112). Under normal conditions, estrogen inhibits osteoclastogenesis and maintains a relative balance of the RANKL/OPG system; after estrogen deficiency, RANKL-mediated osteoclast differentiation is enhanced, and bone resorption is significantly accelerated (113). Concurrently, estrogen deficiency can enhance the activity of the sympathetic nervous system, increasing norepinephrine release, thereby further promoting bone resorption and inhibiting bone formation.

Immune inflammatory amplification is another key link in the development of PMOP. Postmenopausal women often exhibit a state of chronic low-grade inflammation, characterized by a decrease in Treg cells, an increase in Th17 cells, and elevated levels of pro-inflammatory cytokines such as TNF-α, IL-1β, and IL-6 (114). These inflammatory factors can directly promote osteoclastogenesis and also accelerate bone loss by regulating the RANKL/OPG ratio, inducing macrophage polarization towards the M1 type, and enhancing local inflammatory responses (115). Furthermore, gut microbiota dysbiosis and changes in its metabolites may also participate in the immune regulation of PMOP by modulating the Treg/Th17 balance (116). Therefore, PMOP can be summarized as a neuro-immune-bone axis imbalance phenotype primarily driven by “estrogen deficiency-induced immune inflammatory amplification,” accompanied by sympathetic nerve activation and gut microbiota changes.

Within the framework of the neuro-immune-bone axis, PMOP can form a mutually reinforcing process of “estrogen deficiency—sympathetic nerve activation—immune inflammatory amplification—osteoclast activation.” Preclinical studies have shown that modulating inflammatory factors, RANKL signaling, or oxidative stress can improve bone mass and bone microstructure in ovariectomized models (117). However, the clinical maturity of these targets varies: RANKL inhibition has formed a clinical anti-osteoporosis treatment represented by Denosumab, while the application of direct anti-inflammatory, sympathetic nerve regulation, and oxidative stress targeting strategies in PMOP is still mainly in the stages of mechanistic research, animal experiments, or early translation. Therefore, the bone-protective effects of different mechanistic targets in animal models cannot be directly extrapolated to clinical efficacy.

Strategies such as the combination of beta-blockers and anti-inflammatory interventions are currently mechanism-driven exploratory ideas and lack prospective randomized trials and fracture endpoint evidence for PMOP patients (118). At present, the treatment of PMOP should still be based on anti-resorptive or bone-forming drugs recommended by guidelines, combined with calcium and vitamin D management, exercise, and fall prevention. Sympathetic nerve regulation, direct anti-inflammatory therapy, oxidative stress intervention, and gut microbiota modulation are more suitable as future research directions for phenotype stratification and clinical trial validation, rather than being described as established routine combination therapy (119).

### 5.2 Senile osteoporosis

Senile osteoporosis is closely related to multi-system degenerative changes associated with aging. Compared with PMOP, senile osteoporosis is not primarily driven by a single hormone withdrawal but is the result of the combined effects of chronic low-grade inflammation, neurodegeneration, osteoblast senescence, decreased bone marrow stromal cell function, and bone marrow microenvironment degeneration (120, 121). Inflammaging is one of its important features, characterized by persistently elevated levels of pro-inflammatory cytokines such as IL-6 and TNF-α, accompanied by immune cell functional remodeling (122). These inflammatory signals can enhance osteoclast activity, inhibit osteoblast differentiation, and impair bone repair capacity, thereby shifting bone remodeling towards a state of low formation or high resorption (123).

Neurodegenerative changes are also an important component of senile osteoporosis. With aging, sensory nerve function declines, and the release of bone-protective neuropeptides such as CGRP decreases, which may weaken osteogenic support, neurovascular coupling, and bone repair capacity (124). Concurrently, relatively enhanced sympathetic nerve activity or disturbances in neurotransmitter metabolism can further promote RANKL-dependent bone resorption and disrupt the bone marrow microenvironment (125). Additionally, decreased central neuroendocrine regulation, such as hypothalamic-pituitary-target gland axis hypofunction, may also affect the balance between the growth hormone/insulin-like growth factor-1 axis, stress hormones, and bone metabolism (126). Therefore, senile osteoporosis is more characterized by a cumulative dysregulation involving “inflammatory aging, neurodegeneration, and osteogenic decline” rather than a single mechanism of osteoclast activation.

From the perspective of the neuro-immune-bone axis, the intervention focus for senile osteoporosis should not be limited to inhibiting bone resorption but should also include improving the bone marrow microenvironment, maintaining neuro-vascular-bone coupling, reducing chronic low-grade inflammation, and enhancing osteogenic repair capacity (127, 128). Basic interventions such as exercise, nutritional support, and vitamin D and calcium supplementation have strong clinical relevance and can exert comprehensive effects by improving musculoskeletal function, reducing fall risk, and modulating the inflammatory state (129, 130). Some anti-inflammatory drugs, traditional Chinese medicine formulas, or interventions related to the brain-gut-bone axis have shown potential in preclinical models, but their therapeutic efficacy against osteoporosis still requires validation through high-quality clinical research (131). Consequently, managing senile osteoporosis necessitates a multidimensional strategy integrating foundational anti-osteoporosis therapy, exercise and nutritional interventions, and comprehensive chronic disease management.

### 5.3 Secondary osteoporosis

Secondary osteoporosis is bone loss and deterioration of bone quality caused by underlying diseases, long-term drug exposure, or special physiological/pathological conditions, and its pattern of neuro-immune-bone axis imbalance exhibits a stronger dependence on specific etiologies. Diabetes mellitus-related osteoporosis is a typical example (132). Long-term hyperglycemia can affect bone quality through the accumulation of advanced glycation end products, oxidative stress, microvascular damage, and neuropathy; concurrently, hyperglycemia and abnormal insulin/IGF-1 signaling can inhibit osteoblast function and lead to a decline in bone matrix quality (133, 134). Diabetic peripheral neuropathy can also lead to impaired sensory nerve function and reduced CGRP release, thereby weakening local bone protection (135). Concurrently, diabetes-related chronic low-grade inflammation can promote elevated levels of inflammatory factors such as TNF-α and IL-6, further enhancing osteoclast activity and inhibiting bone formation. Therefore, diabetes-related osteoporosis is more akin to a secondary bone loss phenotype driven by “metabolic abnormalities-neuropathy-chronic inflammation-declining bone quality.”

Glucocorticoid-induced osteoporosis is another common type of secondary osteoporosis. Long-term or high-dose glucocorticoid exposure can directly inhibit osteoblast proliferation, differentiation, and survival, reduce bone matrix synthesis, and promote osteocyte apoptosis; it can also enhance bone resorption and decrease bone formation by upregulating RANKL, downregulating OPG, and affecting calcium and phosphorus metabolism (136, 137). It is important to note that glucocorticoids themselves have immunosuppressive effects, so it is not appropriate to simply describe them as “promoting the release of pro-inflammatory cytokines.” The key to their bone loss effect lies in directly inhibiting osteogenesis, disrupting osteocyte homeostasis, altering the RANKL/OPG balance, and acting in concert with the underlying disease inflammation, immobilization, nutritional status, and cumulative dose. Studies have shown that cumulative glucocorticoid dose is an important risk factor for decreased lumbar spine bone mineral density and vertebral fractures in children and adolescents with secondary osteoporosis (138). Therefore, clinical management should strictly evaluate the indications, dose, and duration of glucocorticoid use, and implement bone health monitoring and preventive interventions early.

Osteoporosis related to chronic inflammatory diseases reflects the combined influence of primary disease inflammation, drug exposure, immobilization, malnutrition, and neuro-immune-bone axis abnormalities (139). Active phases of diseases such as rheumatoid arthritis and inflammatory bowel disease can be accompanied by elevated levels of inflammatory factors like TNF-α, IL-1β, IL-6, and RANKL, promoting osteoclast differentiation and bone resorption; long-term pain, reduced activity, muscle loss, impaired nutrient absorption, and glucocorticoid use can further exacerbate bone loss (140). Effective control of the primary disease inflammation can help slow bone loss, but this constitutes merely an “indirect bone protection after controlling the underlying disease” and cannot replace standard anti-osteoporosis treatment. For patients at high risk of fracture, bone mineral density assessment, calcium and vitamin D management, and anti-resorptive or bone-forming therapy should still be implemented according to guidelines, combined with individualized intervention based on the characteristics of the primary disease (141, 142).

Overall, secondary osteoporosis is not driven by a single mechanism; its neuro-immune-bone axis imbalance often depends on the type of primary disease and treatment exposure. Metabolic abnormalities and neuropathy are more prominent in diabetic patients; osteogenesis inhibition and osteocyte damage predominate in those with glucocorticoid exposure; and inflammation factor- and RANKL-driven bone resorption are most evident in patients with chronic inflammatory diseases. Therefore, the prevention and treatment of secondary osteoporosis should be based on controlling the primary disease, reducing avoidable drug-induced bone damage, and implementing standard anti-osteoporosis treatment, while neuro-immune-bone axis-related indicators can serve as important directions for future risk stratification and mechanistic research (**Table 1**).

**Table 1:** Comparison of neuro-immune-bone axis imbalance characteristics in different types of osteoporosis.

Osteoporosis type	Core driving factors	Characteristics of neuro-immune- bone axis imbalance	Main bone metabolism manifestations	Clinical and translational implications	Reference
Postmenopausal osteoporosis	Estrogen Deficiency	Sympathetic nerve activation; decreased Tregs and increased Th17 cells; elevated TNF-α, IL-1β, and IL-6; and RANKL/OPG imbalance.	Osteoclast activation, enhanced bone resorption, rapid bone mass decline.	Standard anti-osteoporosis treatment as a basis; targeting inflammation, RANKL, oxidative stress, and sympathetic regulation can serve as combined intervention directions.	(111–119)
Senile osteoporosis	Aging-related Multisystem Degeneration	Inflammaging; reduction of bone-protective neuropeptides such as CGRP; osteoblast senescence; bone marrow microenvironment degeneration.	Decreased osteogenic capacity, impaired bone repair, bone remodeling imbalance.	Prioritize exercise, nutrition, vitamin D/calcium supplementation, and chronic disease management; note that anti-inflammatory and brain-gut-bone axis interventions require further clinical validation.	(122–130)
Diabetes- related osteoporosis	Hyperglycemia and Metabolic Abnormalities	AGEs accumulation, oxidative stress, and microvascular damage; peripheral neuropathy; decreased CGRP release; chronic low-grade inflammation.	Suppressed osteogenesis, decreased bone matrix quality, increased bone fragility.	Blood glucose and metabolic control are fundamental; neuropathy and inflammatory markers can be used for future risk stratification	(133–135)
Glucocorticoid-induced osteoporosis	Long-term or High-dose Glucocorticoid Exposure	Direct inhibition of osteoblast function; induction of osteocyte apoptosis; upregulation of RANKL and downregulation of OPG; and synergistic effects with underlying diseases and immobilization.	Decreased bone formation, impaired osteocyte homeostasis, increased fracture risk.	Assess glucocorticoid dosage and treatment duration; implement early bone mineral density monitoring and preventive interventions.	(136–138)
Chronic inflammatory disease-related osteoporosis	Primary Disease Inflammation, Drug Exposure, Pain, and Immobilization	Elevated levels of TNF-α, IL-1β, IL-6, and RANKL; persistent immune- inflammatory activation; and bone loss exacerbated by sarcopenia and malnutrition.	Enhanced osteoclast differentiation, increased bone resorption, decreased local and systemic bone mass.	Controlling primary disease inflammation has indirect bone-protective effects; patients with high fracture risk still require standard anti-osteoporosis treatment.	(139–142)

## 6 Therapeutic strategies targeting the neuro-immune-bone axis

**Figure 5:**
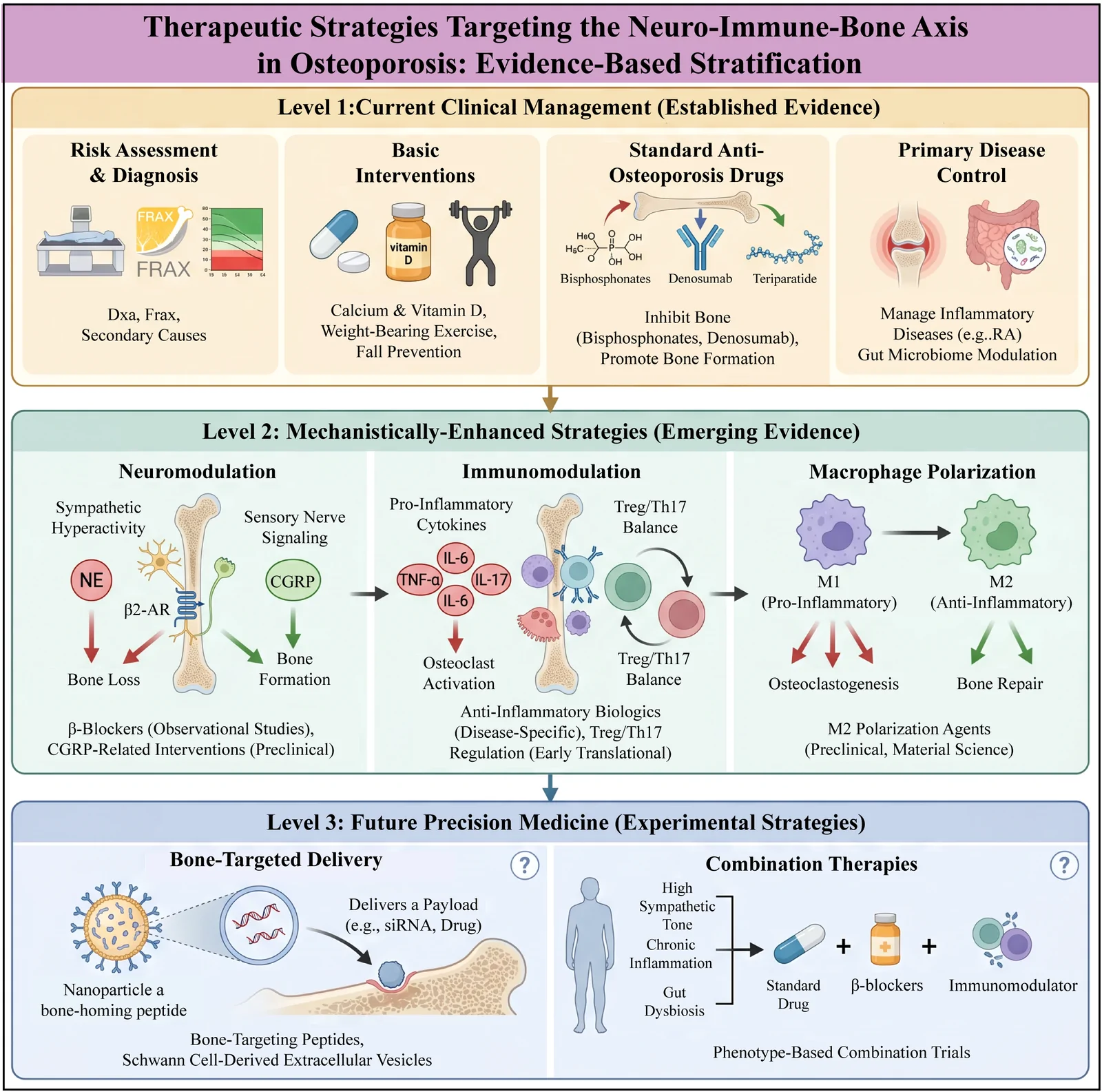
Evidence stratification framework for interventions targeting the neuro-immune-bone axis in osteoporosis. Level 1: Current clinical management. This category encompasses DXA/FRAX assessment, calcium and vitamin D management, exercise, fall prevention, primary disease control, and guideline-recommended medications, all supported by robust clinical evidence. Level 2, Disease-specific or exploratory interventions: includes anti-inflammatory biologics, sympathetic nervous system modulation, CGRP-related interventions, Treg/Th17 regulation, and macrophage remodeling. Except for anti-inflammatory biologics used for specific inflammatory diseases, most are in observational, preclinical, or early translational stages. Level 3, Experimental precision interventions: includes bone-targeted delivery, extracellular vesicles, bone-homing peptides, and phenotype-oriented combination therapies, currently mainly used for mechanistic and technical research, requiring further validation of efficacy and safety. Abbreviations: CGRP, calcitonin gene-related peptide; DXA, dual-energy X-ray absorptiometry; FRAX, Fracture Risk Assessment Tool; Th17, T helper 17 cells; Treg, regulatory T cells.

Targeting the neuro-immune-bone axis provides a new mechanistic perspective for osteoporosis treatment, but the maturity of evidence for different intervention strategies varies considerably. Standard anti-osteoporosis medications, exercise, nutrition, and fall prevention remain the foundation of current clinical management, while strategies such as beta-blockers, CGRP-related interventions, Treg/Th17 regulation, macrophage polarization, and neural-targeted delivery remain largely confined to observational studies, animal experiments, or early translational stages (143). To avoid equating mechanistic plausibility with clinical efficacy, this review categorizes relevant interventions into three categories: Category 1 comprises routine management strategies with guideline recommendations or sufficient clinical trial evidence; Category 2 encompasses disease-specific strategies already used for certain inflammatory diseases but without an independent indication for osteoporosis; and Category 3 consists of exploratory strategies primarily based on observational studies, animal experiments, biomaterials, or early translational research. Therefore, while discussing therapeutic potential, this chapter focuses on distinguishing the evidence sources, clinical indications, and translational stages of different interventions, thereby preventing the mischaracterization of experimental strategies as mature osteoporosis treatment regimens (**Figure 5**).

### 6.1 Neural regulation interventions

Neural regulation interventions leverage the regulatory influence of the sympathetic and sensory nervous systems on bone remodeling. Excessive sympathetic activation can promote RANKL expression, enhance osteoclastogenesis, and inhibit osteoblast function through the NE-β2-AR signaling pathway (144). Therefore, beta-blockers have been proposed as a candidate strategy for intervening in the sympathetic nervous system-bone axis. Some observational studies suggest that long-term users of beta-blockers may have higher bone mineral density or a lower risk of fractures (145). However, this evidence mainly comes from retrospective or observational studies, which are susceptible to confounding factors such as indication bias, underlying cardiovascular disease, concomitant medications, and lifestyle. Thus, the existing results are more hypothesis-generating and do not establish a direct causal anti-osteoporotic effect of beta-blockers. In this review, beta-blockers should be positioned as exploratory drug repurposing candidates for the sympathetic nervous system-bone axis, rather than a clinical treatment recommendation for osteoporosis; their potential skeletal benefits, applicable phenotypes, and risk-benefit ratio still require validation through prospective clinical trials.

Sensory nerve-related interventions primarily focus on CGRP and nerve regeneration. In animal models, CGRP can promote osteoblast function, inhibit osteoclastogenesis, and improve bone microarchitecture (146). Strategies based on CGRP delivery, sensory nerve repair, or neurovascular regeneration have shown potential in osteoporotic fracture repair and local bone regeneration models. However, current evidence is predominantly derived from small animal models, biomaterial applications, and local delivery studies, and lacks human safety data, dose-ranging studies, long-term anti-fracture outcomes, and evidence of systemic efficacy (147). Therefore, CGRP delivery, sensory nerve regeneration, and neurovascular coupling reconstruction are currently preclinical strategies in the field of local bone repair, with no evidence supporting their use for routine systemic osteoporosis treatment.

Neurotransmitter-related drugs also require special attention regarding bone metabolism risks. Selective serotonin reuptake inhibitors can affect serotonin signaling, which has both central and peripheral effects on bone metabolism. Long-term SSRI use has been associated with decreased bone mineral density and increased fracture risk in some studies, indicating that they should not be regarded merely as a mechanism to modulate neurotransmitters for improving bone metabolism (148, 149). Similarly, drugs affecting sympathetic nervous system signaling, such as β2-AR agonists, may also have adverse effects on bone metabolism. Future preclinical research could focus on evaluating whether bone-targeted or local delivery can reduce the off-target effects and safety risks associated with systemic neural signaling modulation. For example, Schwann cell-derived extracellular vesicles or bone-targeting peptides may improve local delivery efficiency, but their pharmacokinetics, immunogenicity, manufacturing standards, and long-term safety still require systematic validation (150).

### 6.2 Immunomodulatory strategies

The core of immunomodulatory strategies is to reduce pro-inflammatory cytokine-driven osteoclast activation and restore bone immune microenvironment homeostasis. Inflammatory cytokines such as TNF-α, IL-1β, IL-6, and IL-17 can promote osteoclastogenesis through the RANKL/OPG system, NF-κB/MAPK, and JAK/STAT pathways, so anti-inflammatory biologics have a certain bone-protective significance in inflammatory bone loss (151). In patients with inflammatory diseases such as RA, TNF-α inhibitors, IL-6 receptor antagonists, and IL-17 inhibitors can indirectly maintain or improve bone mineral density by controlling primary disease activity, reducing inflammatory cytokine levels, and decreasing osteoclast signaling (152). It should be clarified that the clinical application of these drugs is based on controlling the primary inflammatory disease, not treating primary osteoporosis. Their skeletal benefits should be considered indirect effects of disease control and should not be extrapolated to support an independent anti-osteoporosis indication or replace guideline-recommended anti-resorptive or bone-forming therapies.

Restoring the Treg/Th17 balance is another important direction for immunomodulation. Treg cells can inhibit osteoclastogenesis through factors such as IL-10 and TGF-β, while Th17 cells and their secreted IL-17A can promote RANKL expression and osteoclast activation (152, 153). In some osteoporosis patients and animal models, the Treg/Th17 balance shifts toward Th17 dominance, suggesting its potential involvement in inflammation-driven bone loss. Strategies such as low-dose IL-2, IL-17 inhibition, mesenchymal stem cell exosomes, and gut microbiota modulation have been explored to restore the Treg/Th17 balance (154, 155). However, current evidence is primarily derived from studies on inflammatory diseases, animal models, or early translational work, and lacks high-quality clinical trials evaluating endpoints such as fragility fractures, long-term bone mineral density changes, and safety in osteoporosis patients. Therefore, Treg/Th17 regulation should be positioned as an exploratory, phenotype-oriented immune intervention strategy, and no clinical protocol for routine osteoporosis management has been established yet. Its near-term value may lie more in immune phenotype identification, mechanism validation, and clinical trial population enrichment, rather than direct therapeutic application.

Macrophage polarization regulation is also considered a potential strategy for improving the bone immune microenvironment. M1-type macrophages release TNF-α, IL-1β, and IL-6, promoting osteoclastogenesis and bone resorption; M2-type macrophages secrete IL-10, TGF-β, and other factors, facilitating inflammation resolution and tissue repair (156, 157). Some preclinical studies have shown that IL-4-loaded materials, miRNA regulation, nanoplatforms, and extracellular vesicles can promote M2 polarization or inhibit M1 responses, thereby improving bone defect repair or reducing inflammatory bone loss (158, 159). However, the M1/M2 classification is a simplified description of the complex macrophage functional spectrum, and *in vivo* macrophage states are continuous, dynamic, and time-dependent. Existing intervention studies are still mainly focused on animal models, biomaterials, and local drug delivery, lacking standardized targets, unified phenotypic indicators, human safety data, and clinical evidence with fragility fractures as endpoints.

It should also be noted that certain drugs that promote M2-related phenotypes, such as PPARγ agonists, may simultaneously inhibit osteoblast differentiation or increase fracture risk (160). Therefore, macrophage polarization does not simply produce bone protection through “inhibiting M1 and promoting M2”; related research should simultaneously evaluate inflammatory status, osteogenesis, osteoclastogenesis, bone quality, and systemic metabolic effects. Overall, research into macrophage polarization remains largely confined to animal models, biomaterials, and drug delivery systems. Due to the absence of standardized intervention targets, long-term human safety data, and anti-fracture outcome evidence, it cannot currently be recommended as an immunotherapy for osteoporosis.

### 6.3 Exercise, mechanical manipulation, and bone-targeted comprehensive therapy

Compared with neural and immune-targeted therapies still in the exploratory stage, exercise, nutrition, standard anti-osteoporosis medications, and fall prevention remain the most clinically operable foundations for comprehensive treatment (161). Weight-bearing exercise, resistance training, and balance training can promote bone formation through mechanical stimulation, improve muscle strength and postural control, reduce fall risk, and may indirectly improve neuro-immune-bone axis homeostasis by modulating sympathetic nerve activity, sensory nerve signaling, and chronic low-grade inflammation (162). Therefore, exercise intervention is not only a local mechanical stimulus for bone but also a systemic intervention integrating muscle, neural, immune, and skeletal metabolism.

For patients unable to perform adequate routine exercise due to frailty, pain, neuromuscular dysfunction, or high fall risk, practical interventions based on exogenous mechanical stimulation may serve as individualized adjunctive measures. Manual therapy, massage, joint mobilization, and other mechanical stimuli may influence the bone and joint microenvironment through mechanoreceptors, sensory nerve activation, and local neuroimmune modulation (163). Recent reviews and some early human studies suggest that such interventions may improve pain, functional status, or neuroimmune responses, but direct evidence of benefits on bone mineral density, bone turnover, and fragility fracture risk remains insufficient (96). Therefore, mechanical manipulation can be used as an exploratory non-pharmacological supplement for patients with limited mobility but cannot replace feasible exercise rehabilitation, standard anti-osteoporosis medications, and fall prevention. Its application should consider the patient’s fracture risk, pain level, functional status, and comorbidities, and avoid excessive mechanical loading on severely osteoporotic or recently fractured sites.

Nutritional intervention is also a fundamental component of osteoporosis management. Calcium and vitamin D supplementation supports bone mineralization and calcium-phosphorus metabolism and is one of the basic measures for osteoporosis prevention and treatment. Vitamin D also exhibits immunomodulatory effects, influencing Treg/Th17 balance and inflammatory responses (164). Nutrients such as ω-3 polyunsaturated fatty acids have anti-inflammatory potential and can improve the bone microenvironment under low-grade inflammation by reducing levels of pro-inflammatory cytokines like TNF-α and IL-6 (165). However, nutritional interventions should generally be part of comprehensive management, not a substitute for standard anti-osteoporosis medications.

In terms of pharmacotherapy, bisphosphonates, denosumab, and bone-forming agents remain the core treatment options for patients at high fracture risk (166). Among them, denosumab inhibits osteoclastogenesis by blocking RANKL-RANK signaling, bisphosphonates primarily inhibit osteoclast-mediated bone resorption, and teriparatide promotes bone formation through intermittent PTH signaling (167). The limitations, safety, and sequential management issues of these drugs will be further discussed in the next section.

In the future, on the basis of standard treatment, mechanism-enhanced combination interventions could be explored depending on whether patients have conditions such as sympathetic hypertonia, chronic inflammation, sensory nerve degeneration, gut microbiota dysbiosis, or secondary disease backgrounds. However, current evidence on combining standard drugs with beta-blockers, CGRP delivery, or immunomodulators is still insufficient and not suitable for routine recommendation. Mechanical manipulation should also be positioned as an adjunctive non-pharmacological intervention for patients with limited mobility, rather than an independent anti-osteoporosis therapy.

### 6.4 Limitations and safety considerations of guideline-recommended anti-osteoporosis drugs

Although bisphosphonates, denosumab, and bone-forming agents can significantly reduce the risk of fragility fractures, their clinical application remains constrained by adverse reactions, administration routes, compliance, treatment duration restrictions, and post-discontinuation sequential management. Therefore, drug selection should comprehensively consider fracture risk, renal function, serum calcium levels, cardiovascular risk, prior treatment history, and patient preferences, rather than being based solely on bone mineral density.

Bisphosphonates are commonly used anti-resorptive drugs, but oral formulations may cause upper gastrointestinal discomfort or esophageal irritation and are subject to strict administration and compliance requirements; intravenous formulations may cause transient acute-phase reactions, and caution is needed in patients with renal impairment. Long-term treatment is also associated with rare but serious atypical femoral fractures and osteonecrosis of the jaw (168). Therefore, the benefits of treatment should be regularly assessed based on the patient’s ongoing fracture risk, treatment duration, and individual risk factors to determine whether to continue treatment or implement a drug holiday.

Denosumab effectively inhibits RANKL-RANK signaling but may cause hypocalcemia, particularly in patients with severe renal impairment, requiring pre-treatment assessment and post-treatment monitoring (169). Although osteonecrosis of the jaw and atypical femoral fractures are rare, they should still be considered. Critically, denosumab should not be delayed or discontinued without a subsequent plan, as bone turnover may rebound rapidly after discontinuation, increasing the risk of multiple vertebral fractures (170). Therefore, a long-term administration and anti-resorptive sequential treatment plan should be established before initiating therapy.

Bone-forming agents such as teriparatide, abaloparatide, and romosozumab are primarily indicated for patients at very high fracture risk, but their use is constrained by the requirement for injectable administration, cost, treatment duration, and safety factors (171). Parathyroid hormone analogs may cause nausea, dizziness, transient orthostatic hypotension, and calcium metabolism abnormalities (172); romosozumab, in addition to potentially causing hypocalcemia and injection site reactions, requires special assessment of potential cardiovascular risks. After completing bone-forming therapy, sequential anti-resorptive treatment is usually needed to maintain the gained bone mass and anti-fracture benefits (173).

Overall, the safety and management limitations of currently recommended drugs do not diminish their central role in osteoporosis treatment, but they highlight the need for greater emphasis on individualized selection, compliance management, and sequential therapy in clinical practice. These unmet needs also provide a basis for developing safer, more precise neural, immune, and bone-targeted strategies. Based on evidence maturity, clinical benefits, and safety, interventions related to the neuro-immune-bone axis can be categorized into three levels: Level 1 includes current basic clinical management, including fracture risk assessment, DXA and FRAX evaluation, exercise, nutritional support, fall prevention, primary disease control, and guideline-recommended drugs; for those unable to exercise adequately, individualized mechanical manipulation may be cautiously used as an adjunctive measure. Level 2 includes mechanism-oriented exploratory interventions, including adjunctive strategies targeting inflammatory burden, sympathetic hypertonia, sensory nerve degeneration, and gut microbiota dysbiosis. Level 3 includes precision combination therapy research based on neuro-immune-bone axis phenotypes. It is important to emphasize that emerging biologics and other experimental strategies should not be presumed to be safer or more effective simply because existing treatments have limitations; they can only be translated into viable individualized treatment options following rigorous validation of long-term safety, anti-fracture efficacy, target populations, and risk-benefit profiles (**Table 2**).

**Table 2:** Osteoporosis treatment strategies and evidence stratification targeting the neuro-immune-bone axis.

Strategy category	Representative strategy	Main mechanism of action	Evidence Maturity	Clinical positioning and considerations	Reference
Sympathetic nervous system modulation	Beta-blockers; β2-AR signaling regulation	Inhibits NE-β2-AR- mediated RANKL expression and osteoclast activation.	Low to moderate: mainly observational studies	Represents a potential avenue of research for bone loss associated with high sympathetic tone; is not currently established as a conventional anti-osteoporosis therapy.	(144, 145)
Sensory nerve/CGRP intervention	CGRP delivery; sensory nerve repair; neurovascular regeneration	Promotes osteogenesis, inhibits osteoclastogenesis, improves bone repair microenvironment.	Low: mainly animal and materials science studies	More suitable for research on osteoporotic fracture repair or local bone regeneration.	(146, 147)
Bone-targeted and local delivery	Schwann cell-derived extracellular vesicles; bone-targeting peptides; local delivery carriers.	Enhances local enrichment of neural or immunomodulatory factors in bone tissue, reducing systemic off-target effects.	Low: preclinical studies	Requires further clarification of pharmacokinetics, immunogenicity, preparation standards, batch consistency, and long-term safety.	(150)
Anti- inflammatory Biologics	TNF-α, IL-6, IL-17 pathway inhibitors	Reduces inflammatory cytokines and RANKL-related osteoclast signaling.	Moderate: disease- specific clinical evidence	Primarily used for inflammatory diseases such as RA; bone protection is often a secondary benefit of controlling the primary disease.	(151, 152)
Treg/Th17 balance Regulation	Low-dose IL-2; IL-17 inhibition; MSC exosomes; Gut microbiota regulation	Enhances acquired immune tolerance, reduces Th17/IL-17A- mediated osteoclast activation .	Low: preclinical or early translational	Warrants investigation as a candidate strategy for precision interventions in patients exhibiting an immune imbalance phenotype.	(154, 155)
Macrophage polarization regulation	IL-4 loaded materials; miRNA; nanoplatforms; extracellular vesicles	Inhibits M1 pro-inflammatory response, promotes M2 repair phenotype	Low: mainly animal and materials science studies	Suitable for research on bone defect repair and inflammatory bone loss; safety and risk of osteogenesis inhibition need attention.	(156–160)
Basic non- pharmacological management	DXA/FRAX assessment; calcium/ Vitamin D; exercise rehabilitation; fall prevention; primary disease control.	Improves bone mineralization, musculoskeletal function, and fall risk; indirectly modulates the neuro-immune- bone axis.	High: clinical routine	Current foundation of osteoporosis management, applicable to most patients.	(161, 162)
Mechanical manipulation- assisted intervention	Manual therapy; massage; joint mobilization; other exogenous mechanical stimuli.	Influences bone and joint microenvironment through mechanoreception, sensory nerve activation, and local neuroimmune modulation.	Low: mechanistic studies and early human evidence	Can be explored as an adjunctive measure for patients with limited mobility, but cannot replace standard treatment; excessive mechanical loading should be avoided in patients with severe osteoporosis or recent fractures.	(96, 163)
Nutritional and anti- inflammatory adjunctive intervention	Vitamin D; ω-3 polyunsaturated fatty acids, etc.	Improves bone metabolic parameters and reduces chronic low-grade inflammation.	Moderate: substantial adjunctive evidence	Should be a component of comprehensive management; cannot replace standard pharmacotherapy.	(164, 165)
Bisphosphonates	Alendronate; Risedronate; Zoledronic acid, etc.	Inhibits osteoclast function and bony resorption.	High: guideline-recommended with anti-fracture clinical evidence.	May cause gastrointestinal irritation, acute phase reactions, and renal impairment; long-term use requires vigilance for rare atypical femoral fractures and osteonecrosis of the jaw; drug holidays should be individualized.	(168)
Denosumab	RANKL monoclonal antibody	Blocks RANKL– RANK signaling, inhibits osteoclast formation and bony resorption.	High: guideline-recommended with anti-fracture clinical evidence.	Requires vigilance for hypocalcemia and rare osteonecrosis of the jaw and atypical femoral fractures; avoid unplanned discontinuation and promptly initiate sequential anti-resorptive therapy to prevent rebound bone loss and vertebral fractures.	(169, 170)
Bone-forming or dual-action agents	Teriparatide; Abaloparatide; Romosozumab	Promotes bone formation; Romosozumab also inhibits bony resorption.	High: suitable for patients with very high fracture risk.	Limited by injection, cost, and treatment duration; PTH analogs may cause gastrointestinal discomfort, dizziness, and calcium metabolism abnormalities; Romosozumab requires attention to hypocalcemia and cardiovascular risk; sequential anti-resorptive therapy should follow treatment completion.	(171–173)

## 7 Challenges, limitations, and future directions

### 7.1 The causal chain of mechanisms remains unclear

Although the neuro-immune-bone axis provides an important integrative framework for understanding osteoporosis, the key causal chains are still not fully elucidated. Most studies remain at the level of a single system, single cell type, or single animal model, making it difficult to determine the sequence of neural abnormalities, immune imbalance, and bone loss. For example, increased sympathetic nerve activity, decreased sensory neuropeptides, elevated inflammatory cytokines, and osteoclast activation often occur simultaneously during bone loss. However, whether these changes are upstream drivers of osteoporosis or secondary responses to disease progression still lacks sufficient human evidence (174). Furthermore, existing studies primarily rely on *in vitro* co-cultures, gene knockout animal models, or cross-sectional observations in disease models, lacking synchronous analyses of human bone tissue, bone marrow microenvironment, and peripheral nerve-immune status. The local concentrations, receptor distributions, and spatiotemporal release patterns of neurotransmitters and neuropeptides in the bone microenvironment, as well as their dynamic interactions with immune cell subsets, remain difficult to capture in real time (175). Different skeletal sites, such as the vertebrae, femur, mandible, and alveolar bone, also exhibit differences in mechanical load, blood supply, innervation, and immune microenvironment, which may lead to distinct regulatory patterns of the neuro-immune-bone axis at different sites (176, 177). Therefore, future research needs to shift from single-mechanism validation to dynamic causal analyses at multi-cell, multi-timepoint, and multi-tissue levels.

### 7.2 Disease heterogeneity and insufficient classification

Osteoporosis is not a single disease entity but a syndrome of decreased bone strength arising from diverse pathological etiologies. The dominant mechanisms differ among postmenopausal osteoporosis, senile osteoporosis, diabetes-related osteoporosis, glucocorticoid-induced osteoporosis, and inflammatory disease-related osteoporosis. Postmenopausal osteoporosis is more characterized by estrogen deficiency, sympathetic activation, Treg/Th17 imbalance, and RANKL-mediated osteoclast enhancement; senile osteoporosis is characterized by inflammaging, sensory nerve degeneration, bone marrow microenvironment decline, and decreased osteoblast function (178); diabetes-related osteoporosis is often accompanied by hyperglycemia, microvascular damage, neuropathy, and impaired bone matrix quality; glucocorticoid-induced osteoporosis is primarily marked by osteoblast inhibition, osteocyte damage, and RANKL/OPG imbalance; while osteoporosis associated with chronic inflammatory diseases is jointly influenced by primary disease inflammation, immobilization, malnutrition, and drug exposure (179, 180). Therefore, a single neuro-immune-bone axis imbalance pattern cannot simply account for the pathophysiology in all osteoporosis patients. Current research still lacks actionable clinical classification criteria and biomarker panels capable of distinguishing “sympathetic-dominant,” “sensory nerve degeneration-dominant,” “inflammation-dominant,” “metabolic abnormality-dominant,” or “osteoblast decline-dominant” types of osteoporosis. Future studies should establish stratified cohorts across different osteoporosis populations, comprehensively evaluating bone mineral density, bone turnover markers, inflammatory cytokines, neuropeptides, metabolic status, muscle function, and fracture outcomes to identify the dominant links of neuro-immune-bone axis imbalance in different patients.

### 7.3 Insufficient evidence for clinical translation

Therapeutic strategies targeting the neuro-immune-bone axis are theoretically attractive, but most remain at the stage of observational studies, animal experiments, or early translation. The relationship between beta-blockers and bone mineral density or fracture risk is primarily derived from animal studies and observational clinical research, which may still be confounded by indication bias, cardiovascular comorbidities, and concomitant medications, precluding their current recommendation as routine anti-osteoporosis therapy (181). Strategies such as CGRP supplementation, sensory nerve regeneration, hydrogel delivery, and bone-targeted neuropeptide release are primarily used in fracture repair or local bone regeneration models, and their feasibility as systemic anti-osteoporosis therapies still requires validation (182). Immunomodulatory strategies face similar issues. Biologics such as TNF-α, IL-6, and IL-17 inhibitors may indirectly improve bone metabolism in inflammatory diseases like rheumatoid arthritis and psoriatic arthritis, but their bone-protective effects mainly stem from controlling primary disease inflammation and cannot be directly extrapolated to general osteoporosis patients (183, 184). Strategies including low-dose IL-2, Treg modulation, Th17 inhibition, macrophage polarization, miRNA intervention, probiotics, traditional Chinese medicine formulas, and nanomaterials are mostly still in preclinical or early exploratory stages, with their long-term safety, target populations, therapeutic windows, and anti-fracture benefits yet to be clarified (185). Therefore, future translational research should refrain from conflating mechanistic plausibility with clinical efficacy and should instead prioritize evidence level stratification, phenotype enrichment, and prospective clinical trials to ensure rigorous validation.

### 7.4 Opportunities from new technological platforms

Emerging technological platforms offer significant opportunities for dissecting the neuro-immune-bone axis. Single-cell RNA sequencing and spatial omics can resolve the spatial relationships and molecular interactions among bone marrow mesenchymal cells, osteoblasts, osteoclasts, osteocytes, immune cells, and nerve-related cells at high resolution, thereby facilitating the identification of key cell subsets and signaling pathways in different osteoporotic states (186, 187). Neural tracing, tissue clearing, three-dimensional imaging, and *in vivo* imaging techniques facilitate the observation of sensory and sympathetic nerve distribution, remodeling, and their spatial coupling with blood vessels and immune cells in bone tissue (188). Multi-omics integration, including transcriptomics, proteomics, metabolomics, lipidomics, microbiomics, and radiomics, can help construct a more comprehensive disease map of the neuro-immune-bone axis (189). Combined with machine learning algorithms, these data hold promise for identifying potential endotypes, predicting fracture risk, and screening treatment-responsive populations (190). Additionally, bone organoids, microfluidic chips, and multi-cell co-culture systems can recapitulate cellular interactions among neural cells, immune cells, and bone cells *in vitro*, providing highly controlled experimental platforms for drug screening and mechanism validation (191). However, these technologies are currently primarily used for mechanism discovery and are still far from routine clinical classification and treatment decision-making; their standardization, reproducibility, and clinical accessibility require further validation.

### 7.5 Future research pathways

Future research on the neuro-immune-bone axis should shift from isolated mechanistic descriptions to an integrated continuum of “mechanism validation-phenotype identification-translational intervention.” First, a biomarker panel related to the neuro-immune-bone axis should be established, with candidate indicators including sympathetic nerve activity-related markers, neuropeptides such as CGRP and SP, inflammatory cytokines such as TNF-α, IL-6, IL-17A, and IL-10, the Treg/Th17 ratio, the RANKL/OPG ratio, bone turnover markers, and gut microbiota and metabolite profiles. Second, studies utilizing prospective, stratified cohorts of different types of osteoporosis should be undertaken to systematically compare the neuro-immune-bone axis dysregulation patterns in postmenopausal, senile, diabetes-related, glucocorticoid-induced, and inflammatory disease-related osteoporosis. Third, future studies should simultaneously assess bone mineral density, bone microstructure, bone turnover markers, inflammatory cytokines, neuropeptide levels, muscle function, fall risk, and fracture outcomes, rather than depending exclusively on bone mineral density as a single endpoint. Fourth, phenotype-enriched intervention trials should be implemented, specifically including the validation of anti-inflammatory or immunomodulatory strategies in patients with high inflammatory burden, the exploration of CGRP-related local therapies in patients with sensory nerve degeneration or poor fracture repair, and the evaluation of the safety and skeletal benefits of neuromodulation strategies in populations with high sympathetic tone. Finally, multidisciplinary collaboration should be strengthened, integrating orthopedics, endocrinology, immunology, neuroscience, geriatric medicine, and bioengineering to advance the neuro-immune-bone axis from a mechanistic concept to a verifiable, classifiable, and translatable precision intervention framework (**Table 3**).

**Table 3:** Challenges, limitations, and future directions of neuro-immune-bone axis research.

Main issues	Current limitations	Key improvement directions	Research and translational significance	Reference
Unclear mechanistic causal chain	Neurological abnormalities, immune imbalance, and bone loss often occur simultaneously, making it difficult to determine the sequence; most studies are limited to single cells, single models, or cross-sectional analyses.	Perform synchronous analyses across multiple cell types, time points, and tissues; combine human bone tissue, bone marrow microenvironment, and peripheral neuro-immune indicators.	Identify the true upstream drivers and key intervention targets in the neuro-immune-bone axis.	(174–177)
Inadequate characterization of disease heterogeneity and classification	Significant differences in mechanisms among different types of osteoporosis, but currently lacking actionable clinical classifications and biomarker combinations.	Establish stratified cohorts, comprehensively assess bone mineral density, bone turnover markers, inflammation cytokines, neuropeptides, metabolic status, and fracture outcomes.	Categorize patients into subtypes dominated by sympathetic activity, sensory nerve degeneration, inflammation, metabolic abnormalities, or osteogenic decline.	(178–180)
Insufficient clinical translation evidence	Interventions such as beta-blockers, CGRP delivery, Treg/Th17 regulation, macrophage polarization, and nanomaterials remain largely in observational or preclinical stages	Conduct evidence level stratification, phenotype enrichment, and prospective clinical trials; focus on verifying safety, therapeutic window, and anti-fracture benefits.	Avoid equating mechanistic plausibility directly with clinical effectiveness, and promote the translation of experimental strategies into clinically applicable solutions.	(181–185)
Inadequate translation of new technologies	Single-cell omics, spatial omics, neural tracing, organoids, and microfluidic chips are mostly used for mechanism discovery, with insufficient standardization and clinical accessibility	Combine single-cell/spatial omics, multi-omics integration, 3D image reconstruction, machine learning, and *in vitro* simulation platforms.	Identify key cell subpopulations, construct disease maps, predict fracture risk, and screen treatment-responsive populations.	(186, 187, 189)
Need for systematic future research pathways	Most studies are single-point mechanism validations, lacking a continuous pathway from mechanism discovery to clinical classification and interventional trials.	Establish biomarker panels; conduct prospective cohorts for different types of osteoporosis; design phenotype-enriched interventional trials.	Form a translatable research framework of “mechanism validation— classification identification— precision intervention”.	(190, 191)

## 8 Conclusion

Osteoporosis is not merely caused by an imbalance between osteoblasts and osteoclasts, but is a systemic disease involving the nervous system, immune system, and bone remodeling process. Current research suggests that sympathetic overactivation, sensory nerve degeneration, abnormalities in sensory neuropeptides such as CGRP, sustained release of inflammatory cytokines, Treg/Th17 imbalance, and abnormal macrophage polarization may all contribute to bone loss and bone microstructure destruction by affecting the RANKL/OPG system, osteoblast function, and osteoclast activity. The imbalance patterns of the neuro-immune-bone axis differ among various types of osteoporosis. Postmenopausal osteoporosis is more prominently driven by estrogen deficiency-induced immune inflammation amplification and sympathetic activation; senile osteoporosis is characterized by inflammaging, sensory nerve degeneration, and bone marrow microenvironment decline; secondary osteoporosis is often influenced by the combined effects of underlying conditions, drug exposure, metabolic abnormalities, and chronic inflammation. Therefore, mechanism-based classification of the neuro-immune-bone axis may help better explain the clinical heterogeneity of osteoporosis.

Intervention strategies targeting the neuro-immune-bone axis may provide complementary approaches to traditional anti-resorptive or bone-forming therapies, including neuromodulation, immune regulation, exercise and nutritional interventions, and mechanism-oriented combination therapies. However, most strategies such as beta-blockers, CGRP-related treatments, Treg/Th17 regulation, macrophage polarization, probiotics, Chinese medicine compounds, and nanomaterials are still in observational studies, animal experiments, or early translational stages and cannot yet be directly recommended for routine clinical practice. Future research should integrate single-cell omics, spatial omics, bone marrow immune profiling, neuropeptide detection, and long-term fracture outcomes to establish a verifiable classification system for the neuro-immune-bone axis, and evaluate the clinical efficacy of different intervention strategies through prospective cohorts and phenotype-enriched clinical trials, thereby advancing osteoporosis management from uniform treatment toward mechanism-based stratification and individualized intervention.
